# Genome-wide identification of ZmMYC2 binding sites and target genes in maize

**DOI:** 10.1186/s12864-024-10297-z

**Published:** 2024-04-23

**Authors:** Lijun Liu, Yuhan Zhang, Chen Tang, Jine Wu, Jingye Fu, Qiang Wang

**Affiliations:** 1 State Key Laboratory of Crop Gene Exploration and Utilization in Southwest China, College of Agronomy, Sichuan Agricultural University, 611130 Chengdu, China; 2 College of Life Science, Sichuan Agricultural University, 625014 Yaan, China

**Keywords:** Jasmonate, MYC2, Transcription factor, *cis*-element, Maize

## Abstract

**Background:**

Jasmonate (JA) is the important phytohormone to regulate plant growth and adaption to stress signals. MYC2, an bHLH transcription factor, is the master regulator of JA signaling. Although MYC2 in maize has been identified, its function remains to be clarified.

**Results:**

To understand the function and regulatory mechanism of MYC2 in maize, the joint analysis of DAP-seq and RNA-seq is conducted to identify the binding sites and target genes of ZmMYC2. A total of 3183 genes are detected both in DAP-seq and RNA-seq data, potentially as the directly regulating genes of ZmMYC2. These genes are involved in various biological processes including plant growth and stress response. Besides the classic *cis*-elements like the G-box and E-box that are bound by MYC2, some new motifs are also revealed to be recognized by ZmMYC2, such as nGCATGCAnn, AAAAAAAA, CACGTGCGTGCG. The binding sites of many ZmMYC2 regulating genes are identified by IGV-sRNA.

**Conclusions:**

All together, abundant target genes of ZmMYC2 are characterized with their binding sites, providing the basis to construct the regulatory network of ZmMYC2 and better understanding for JA signaling in maize.

**Supplementary Information:**

The online version contains supplementary material available at 10.1186/s12864-024-10297-z.

## Background

To respond the changeable environment, plants evolve complex mechanisms to integrate exogenous environmental stimuli and endogenous plant hormone signaling to coordinate growth and stress response. Jasmonate (JA) is an important phytohormone to widely participate into plant growth, immunity and defense through reprogramming gene expression [1–3]. The biosynthesis of JA is initiated from α-linolenic acid with sequential oxidation and catalysis by LOX, AOS, AOC and other enzymes to form jasmonic acid, finally to generate bioactive JA-Ile by JAR1 [4]. When JA level is elevated in plants, JA-Ile is perceived and bound by the receptor complex consisting of the F-box protein COI1 and the JA signaling repressor JAZ, resulting in degradation of JAZ by the 26S proteasome and releasing of MYC2 to activate downstream gene expression [5–7].

MYC2 is the key transcription factor to regulate JA signaling genes with conserved functions in plants [8]. MYC2 belongs to the basic-helix-loop-helix (bHLH) family, widely involved in plant defense, growth and development, metabolism and signaling transduction. At the C-terminus, MYC2 has the bHLH domain consisting of the basic region with 10–15 aa and the α-HLH-α motif with 40 aa, essential for DNA binding. At the N-terminus, MYC2 has one trans-activation domain for recruiting MED25 and the JID domain for interacting with JAZ, which is required for fine-tuning downstream gene expression [5, 9, 10]. Under the resting state of JA signaling, JAZ interacts with MYC2 and inhibit regulation of downstream target genes. Once JA signaling is activated, JAZ is degraded and MYC2 is released to target downstream genes [10].

MYC2 mediates the core transcriptional regulation of JA signaling to play diverse roles in stress response, specialized metabolism and growth in many plants [11, 12]. MYC2 is involved in many biological processes, such as root growth [13], seed protein accumulation [14], light signaling [15–17], phytohormone signaling crosstalk [18–21]. MYC2 is also involved in many defensive processes against herbivory and pathogen infection [10, 22], as well as drought response [23–25] and salinity response [26–28]. In maize, ZmMYC2 has been characterized [29]. It regulates maize terpenoid phytoalexin metabolism through involving in JA and ethylene signaling [30]. ZmMYC2 was also reported to bind the promoters of benzoxazinoid and volatile terpenoid biosynthetic genes to regulate corresponding metabolites accumulation in response to herbivory [31]. The homologous ZmMYC7 was identified to regulate ZmERF17 to promote maize resistance to *Fusarium graminearum* [32].

To give the better understanding for ZmMYC2 function and regulatory mechanism, the joint analysis through DAP-seq and RNA-seq was conducted to identify ZmMYC2 target genes and binding sites on maize chromosome. Abundant genes were detected in this study to be regulated by ZmMYC2 directly or indirectly. A number of *cis*-elements were also identified as the binding sites of ZmMYC2 for transcriptional regulation. These data in this study could be used for construction of ZmMYC2 regulatory network and further function characterization.

## Results

### RNA-seq analysis of ZmMYC2 regulating genes

**Fig. 1:**
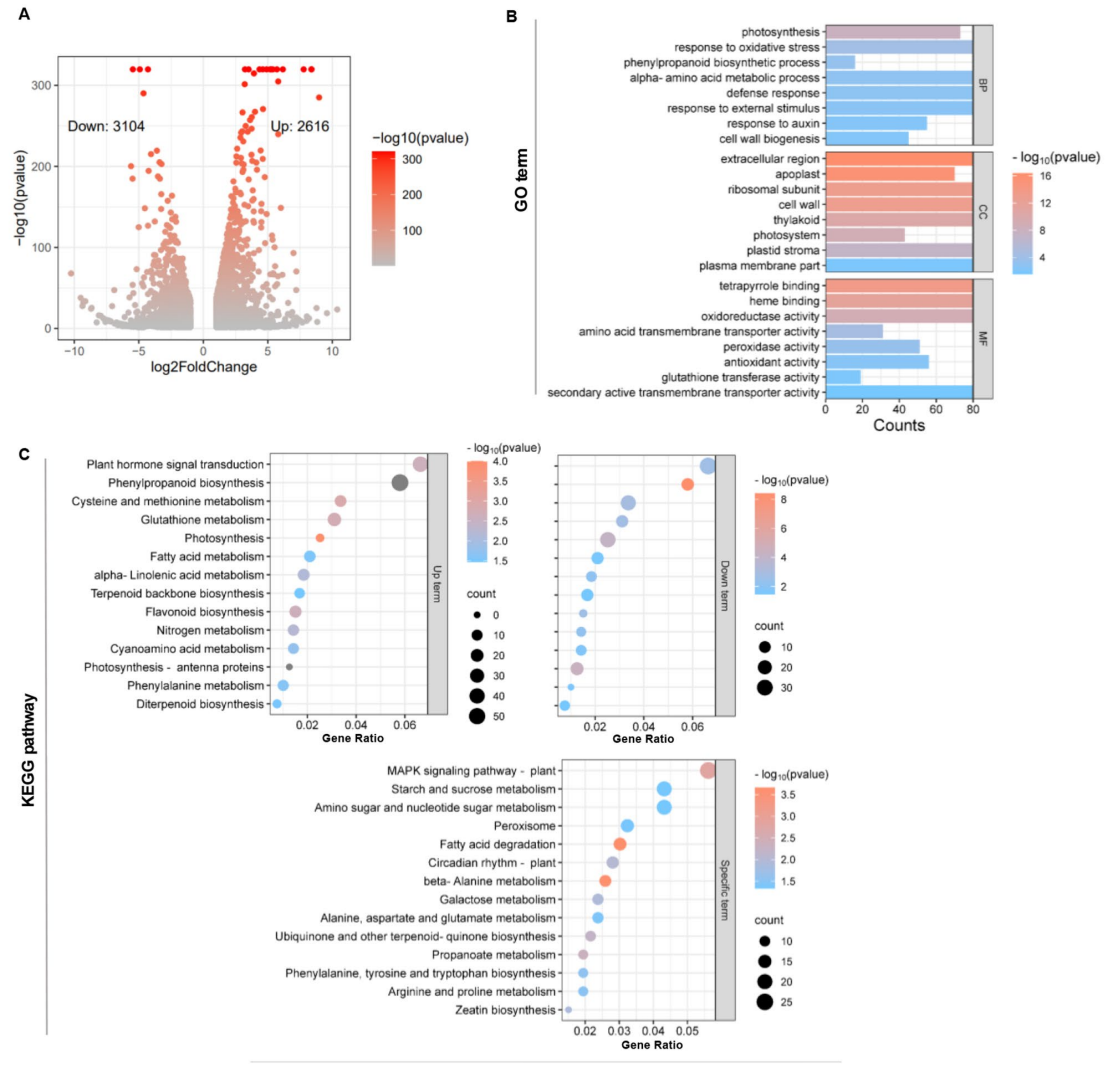
RNA-seq analysis of ZmMYC2 transient overexpression in maize protoplasts. (**A**) volcano plot of DEGs in ZmMYC2 transient overexpression maize protoplasts and the control. Log2Foldchange indicates the fold change of DEGs with upregulation or downregulation. (**B**) GO enrichment analysis of DEGs. BP, biological process. CC, cellular component. MF, molecular function. (**C**) KEGG enrichment analysis of DEGs in different pathways with upregulation (Up term) and downregulation (Down term). The specific enriched pathways only with upregulated DEGs were also shown (Specific term)

Previously we identified ZmMYC2 as the core transcription factor of maize JA signaling [29]. To further characterize ZmMYC2 function and explore the regulating genes, we used maize leaf protoplasts as the convenient and fast platform to transiently overexpress ZmMYC2. The successful transformation and high expression were verified by GFP observation and qPCR validation (Fig S1A-C). The protoplasts with ZmMYC2 overexpression were collected for RNA extraction and RNA-seq analysis. Clear clustering of detecting genes was observed for RNA-seq data as indicated in Fig S1C (Table S1). After overexpression of ZmMYC2 in maize protoplasts, 2616 genes were detected with upregulation and 3104 genes for downregulation (Fig. 1A and Table S2). GO analysis indicated that these DEGs were enriched in various processes such as cell wall, defense, antioxidant activity (Fig. 1B and Table S3). Further KEGG analysis revealed that these DEGs were enriched in many pathways such as plant hormone signal transduction, phenylpropanoid biosynthesis, glutathione metabolism, as well as MAPK cascade, starch and sugar metabolism (Fig. 1C and Table S4). These results suggest that ZmMYC2 exhibits diverse functions.

### Target regulating genes of ZmMYC2

**Fig. 2:**
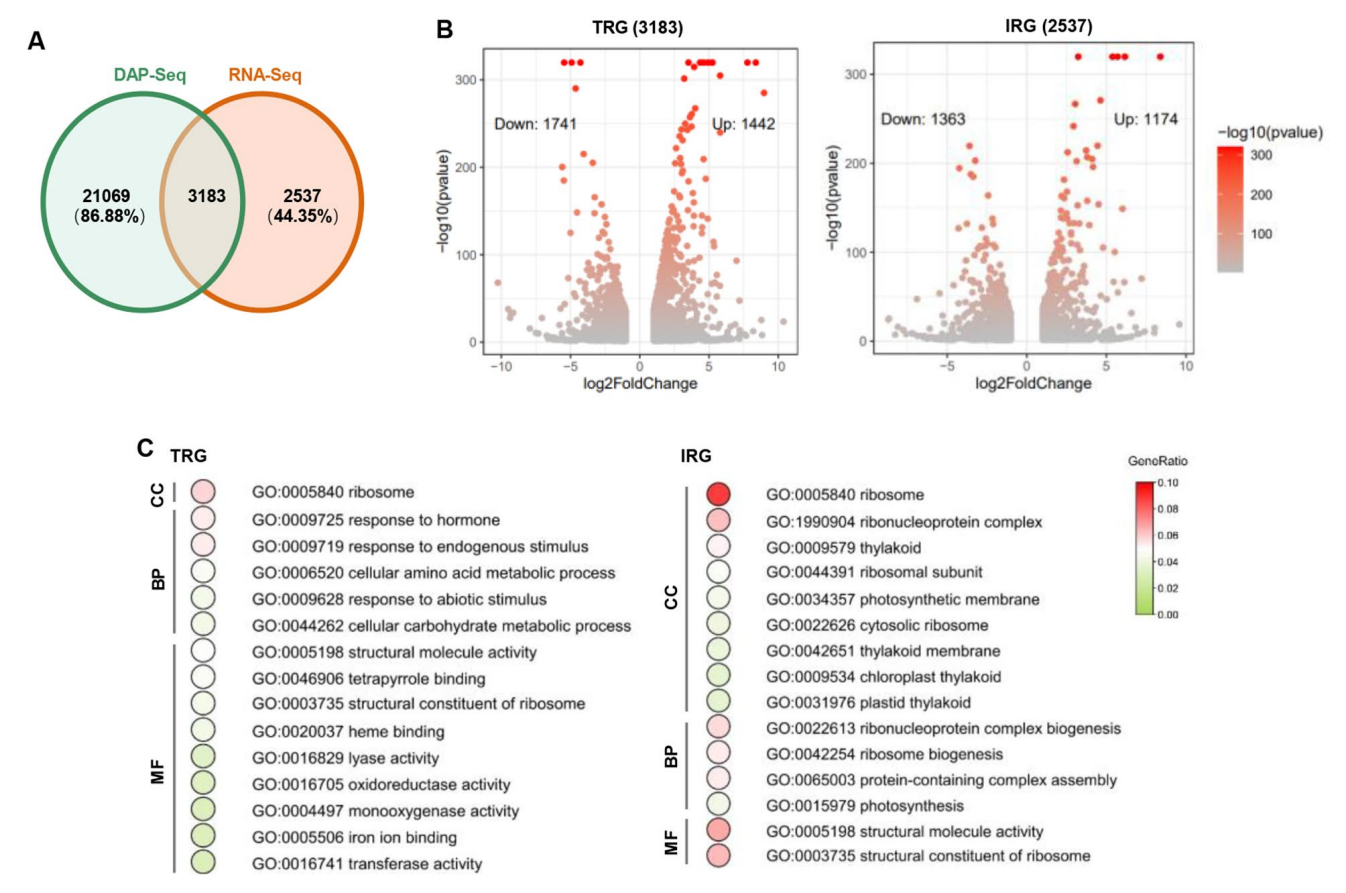
Joint analysis of RNA-seq and DAP-seq for ZmMYC2. (**A**) Venn plot of shared and specific genes by joint analysis of RNA-seq and DAP-seq for ZmMYC2. (**B**) Volcano plots for target regulating genes (TRGs) and indirect regulating genes (IRGs) of ZmMYC2. (**C**) Predominant GO terms of TRGs and IRGs through GO enrichment analysis

We have identified a lot of potential target genes with binding sites using DAP-seq [29]. To explore whether these genes could be regulated by ZmMYC2, we combined the data from DAP-seq and RNA-seq to find the direct target regulating genes of ZmMYC2. From both sequencing data, 3183 genes (Target regulating genes, TRGs) were identified to be bound and regulated by ZmMYC2 (Fig. 2A and Table S5), among which 1741 genes were upregulated and 1442 genes were downregulated by ZmMYC2 (Fig. 2B). In RNA-seq data, expression of other 2537 genes (Indirect regulating genes, IRGs) were observed to be affected by transient overexpression of ZmMYC2 in maize protoplasts, however, they were not detected in DAP-seq data with direct binding, indicating indirect regulation on these genes by ZmMYC2. Among IRG, 1363 genes were upregulated and 1174 genes were downregulated by ZmMYC2 (Fig. 2B). There were also 21, 069 genes to be detected in DAP-seq data with binding by ZmMYC2 but not identified as the DEGs in RNA-seq data (Table S6).

We conducted GO analysis for regulating genes of ZmMYC2 and compared the top 15 GO terms of TRGs and IRGs (Fig. 2C). TRGs are mainly enriched in hormone response, stress response, metabolism and enzymatic activity, indicating that ZmMYC2 directly targets and regulates these genes to play roles in such processes. IRGs are mainly enriched in ribosome, photosynthesis and structure molecule activity, suggesting the indirect role of ZmMYC2 in these processes through regulating IRGs.

### ZmMYC2 is involved in plant growth and stress response

**Fig. 3:**
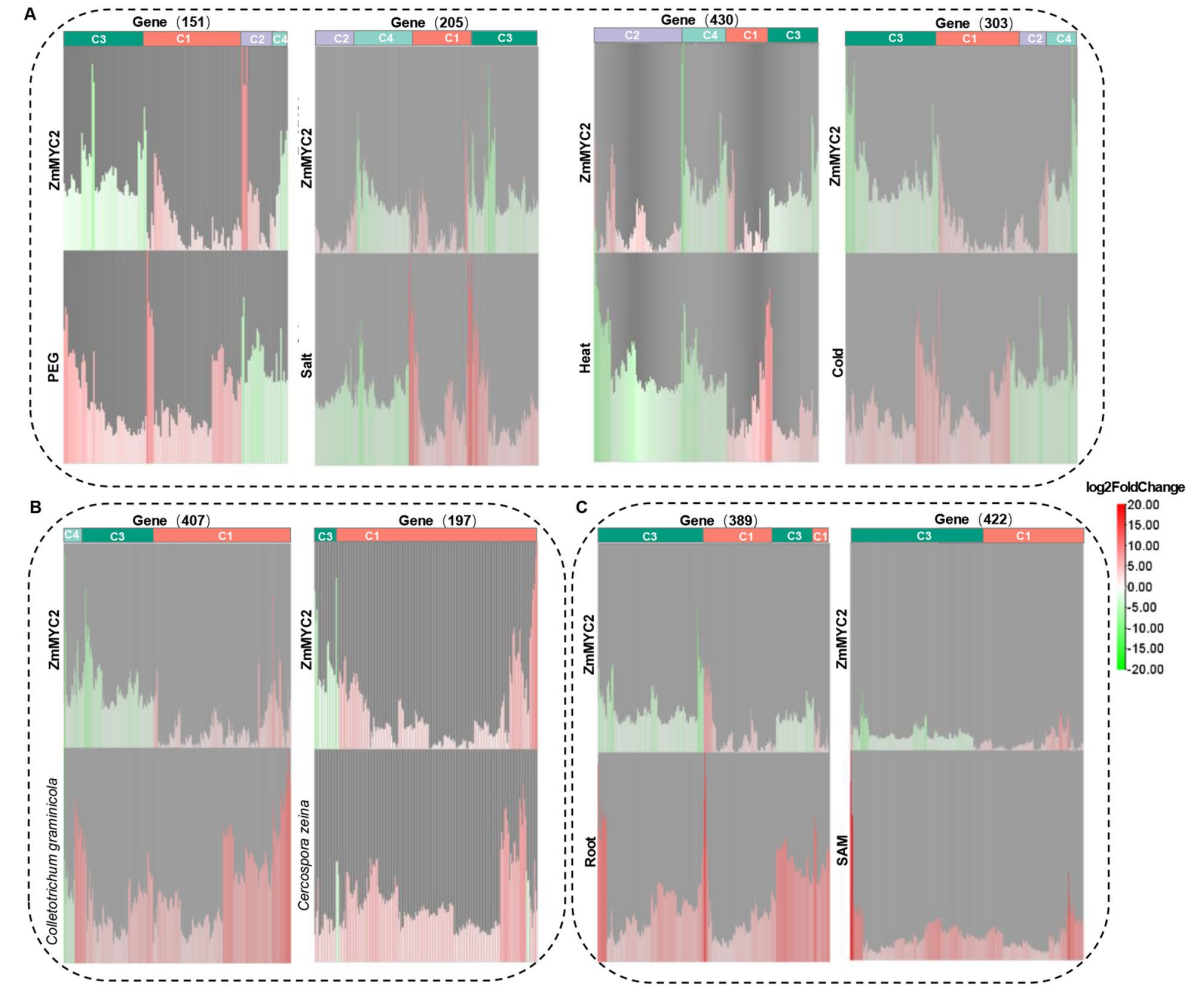
Cluster analysis for gene expression analysis of ZmMYC2 target regulating genes. Gene expression of ZmMYC2 target regulating genes (TRGs) were compared among the RNA-seq data in this study and the transcriptomic data on Maize eFP including root and shoot apical meristem (SAM) growth, treatments of PEG, salt, heat and cold, infection of *Colletotrichum graminicola* and *Cercospora zeina*. (**A**) Cluster analysis of ZmMYC2 TRGs with expression comparison among RNA-seq data in this study and the data with abiotic stress treatments in Maize eFP, biotic stress treatments (**B**), root and SAM growth (**C**). C1-4, clustered genes. C1 and C2 exhibited upregulation by ZmMYC2 but upregulation (C1) or downregulation (C2) in the corresponding treatments. C3 and C4 were repressed by ZmMYC2 but showed increasing (C3) or decreasing (C4) expression in the corresponding treatments

To further analyze the function of ZmMYC2 in regulation of TRGs, we downloaded transcriptomic data related to maize growth and stress response from Maize eFP database for association analysis with our data [33]. 1429 TRGs were screened to be involved in growth and stress response (Table S7), including 303 genes for cold response, 430 for heat, 205 for salinity and 151 for drought by PEG treatment (Fig. 3A and Table S8). In the mean time, 197 and 407 TRGs were observed to respond to *C. zeina* and *C. graminicola* infection, respectively (Fig. 3B and Table S9). 389 and 422 TRGs were involved in root and apical meristem development, respectively (Fig. 3C and Table S10). All these genes were clustered into four categories (C1-4), among which C1 and C2 exhibited upregulation by ZmMYC2 but increased or decreased expression in the corresponding treatments, respectively. Genes in C3 and C4 were repressed by transient overexpression of ZmMYC2 but also showed increased or decreased expression in the corresponding treatments, respectively. All four category genes were detected in abiotic stress responses but C1 and C3 were mainly observed in pathogen infection responses with C1 as the predominant cluster (Fig. 3A-B), suggesting the positive role of ZmMYC2 in pathogen defense. In root and apical meristem development, C1 and C3 were mainly observed but with C3 as the predominant cluster (Fig. 3C), implicating negative roles of ZmMYC2 in growth.

### ZmMYC2 regulates plant hormone related genes

**Fig. 4:**
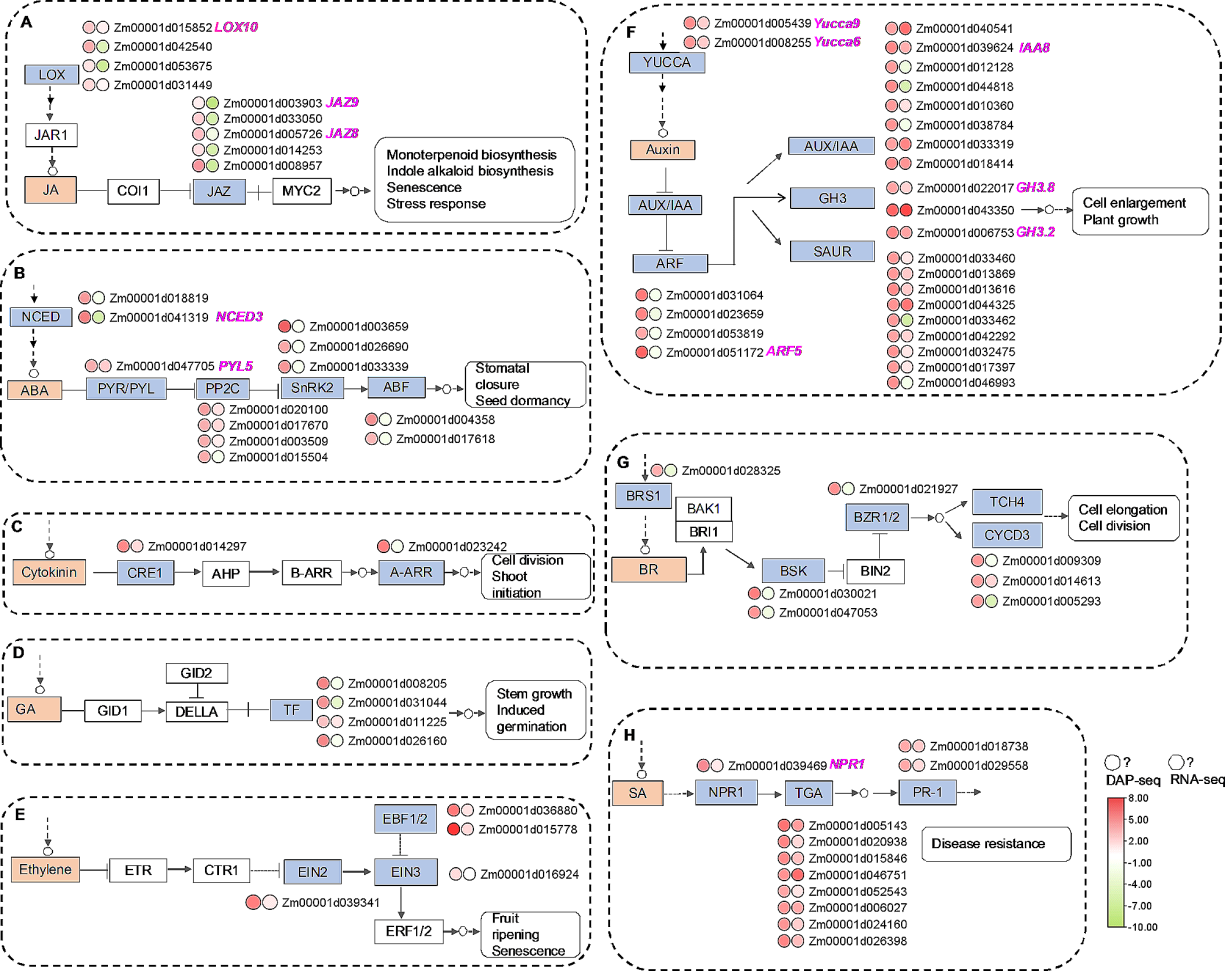
Plant hormone related genes regulated by ZmMYC2. Graphs of eight plant hormone synthesis and signling with key genes shown. (**A**-**H**), Jasmonate (JA), abscisic acid (ABA), cytokinin, gibberellin (GA), ethylene, auxin, brassinolide (BR) and salicylic acid (SA). Plant hormone names are labeled in orange. The key genes regulated by ZmMYC2 as TRGs are highlighted in blue. Circles close to genes indicate fold enrichment in DAP-seq and fold change in RNA-seq data for specific genes, respectively. Value changes are shown by color intensity. The genes with functional characterization were labeled in purple

MYC2 plays the key role in plant hormone signaling crosstalk [10, 34]. Further analysis indicated that ZmMYC2 targeted many genes related to plant hormone biosynthesis and signal transduction (Fig. 4 and Table S11). Among these genes, four types of plant hormones were revealed to be regulated by ZmMYC2 through targeting their biosynthesis genes including JA, abscisic acid (ABA), Auxin and brassinolide (BR) (Fig. 4A-B and F-G). In addition, ZmMYC2 widely regulated plant hormone signaling genes with mainly targeting JAZ, PP2C, SnRK2, ARF, Aux/IAA, BSK and TGA. In these hormone related genes, some have been characterized functionally in maize growth or stress response. For instance, JA related genes, *ZmLOX10* and *ZmJAZ9* were involved in insect resistance, and ZmJAZ8 played roles in defense to *C. graminicola* infection [31, 35, 36]. Auxin biosynthesis genes, *ZmYucca6* and *ZmYucca9* were reported to participate into root geotropism and lodging resistance [37]. Auxin signaling genes, *ZmIAA8*, *ZmGH3.2*, *ZmGH3.8* and *ZmIAA8* were involved in drought resistance and seed development, and *ZmGH3.2* and *ZmGH3.8* with roles in seed aging or leaf senescence, respectively [38–41]. ABA synthesis gene *ZmNCED3* was also identified in drought response and regulated by ZmMYC2 directly here (Fig. 4B) [42].

### ZmMYC2 regulates abundant transcription factors

**Fig. 5:**
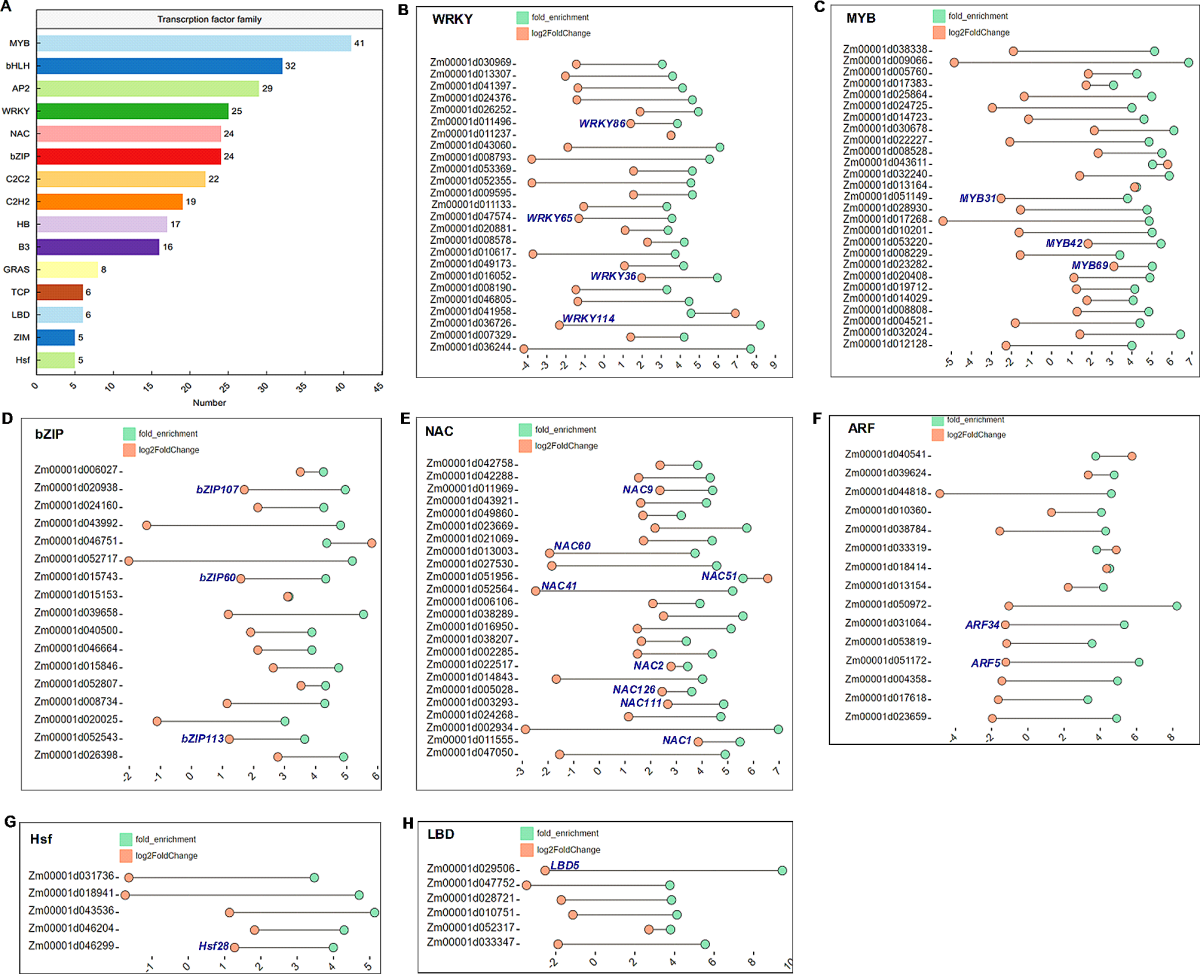
Transcription factors regulated by ZmMYC2. (**A**) Bar plot of transcription factors regulated by ZmMYC2 with families and gene numbers. (**B**-**H**) Lollipop plot of seven transcription factor families regulated by ZmMYC2. Fold enrichment (green circle) by DAP-seq and log2fold change (red circle) by RNA-seq were shown for each genes. The genes highlighted in purple were functional characterized in previous studies

Many transcription factors were also identified as target regulating genes of ZmMYC2 (Fig. 5A and Table S12). Among which, the top three transcription factor families are MYB, bHLH and AP2. We further listed these transcription factors by categories with fold change of gene expression in RNA-seq and enrichment in DAP-seq (Fig. 5B-H). Some of these transcription factors have been characterized functionally (Table 1). For example, overexpression of ZmWRKY114 impaired salinity tolerance [43]. ZmMYB42 negatively regulated lignin synthesis, which was activated by ZmMYB69 to inhibit lignin biosynthesis in maize stem [44, 45]. ZmNAC111 promoted stomotal closure to increase water utilization and drought resistance [46]. ZmARF34 interacted with ZmRUM1 to participate lateral root formation [47]. In these genes, some of them have been identified to respond to JA signal, indicating that ZmMYC2 could mediate such response. For other transcription factors, our results indicated that ZmMYC2 might work upstream to regulate these genes, which might be investigated in the future study.

**Table 1:** Transcription factors regulated by ZmMYC2 with functional characterization

Name	ID	OE/mutant lines		Tested traits	Hormonal response	Function	References
ARF34	Zm00001d031064	-		growth	-	seminal root formation	[47]
ARF5	Zm00001d051172	-		growth	Auxin	root growth and development	[48]
bZIP107	Zm00001d024160	transgenic rice		lead	-	tolerance to Pb stress and decreased Pb absorption	[49]
bZIP113	Zm00001d026398	-		cold	-	Controlling low-temperature germination	[50]
bZIP60	Zm00001d015153	-		heat	-	enhanced expression by heat	[51]
LBD5	Zm00001d029506	mutant maize		drought, growth	ABA, GA	maize growth and the drought response	[52]
MYB31/MYB42	Zm00001d051149	transgenic Arabidopsis		growth	-	decrease in lignin content	[53]
MYB69	Zm00001d023282	transgenic and mutant maize		growth	-	Repress lignin biosynthesis	[45]
NAC1	Zm00001d011555	transgenic Arabidopsis		growth	-	lateral root development	[54]
NAC111	Zm00001d003293	transgenic maize		drought	-	Regulation of growth and anthesis-silking intervals	[46]
NAC126	Zm00001d005028	transgenic *Arabidopsis* and maize		growth	-	Enhanced chlorophyll degradation and promoted leaf senescence,	[55]
NAC2	Zm00001d022517	transgenic Arabidopsis		osmotic	-	seed germination, stomatal closure and ROS scavenging	[56]
NAC41	Zm00001d052564	transgenic rice		pathogen	-	Enhanced resistance to R. solani in rice	[57]
NAC51	Zm00001d051956	-		drought	-	Genome-wide identification and expression pattern	[58]
NAC9	Zm00001d011969	-		low phosphorus	-	Response to low P and regulation in root architecture	[59]
WRKY114	Zm00001d036726	transgenic rice		Salt, growth	ABA, GA	Salt-stress responses and plant height regulation	[43, 60]
WRKY36	Zm00001d049173	-		growth	-	leaf senescence	[61]
WRKY65	Zm00001d047574	transgenic *Arabidopsis*		drought, salt, temperature, pathogen	ABA, SA	ABA, SA-induced	[62]
WRKY86	Zm00001d011496	mutant maize		salt	-	Salt tolerance	[63]
Hsf28	Zm00001d046299	transgenic maize		drought	ABA, JA	Drought resistance	[64]

‘-’means no report

### ZmMYC2 recognizes diverse *cis*-elements

**Fig. 6:**
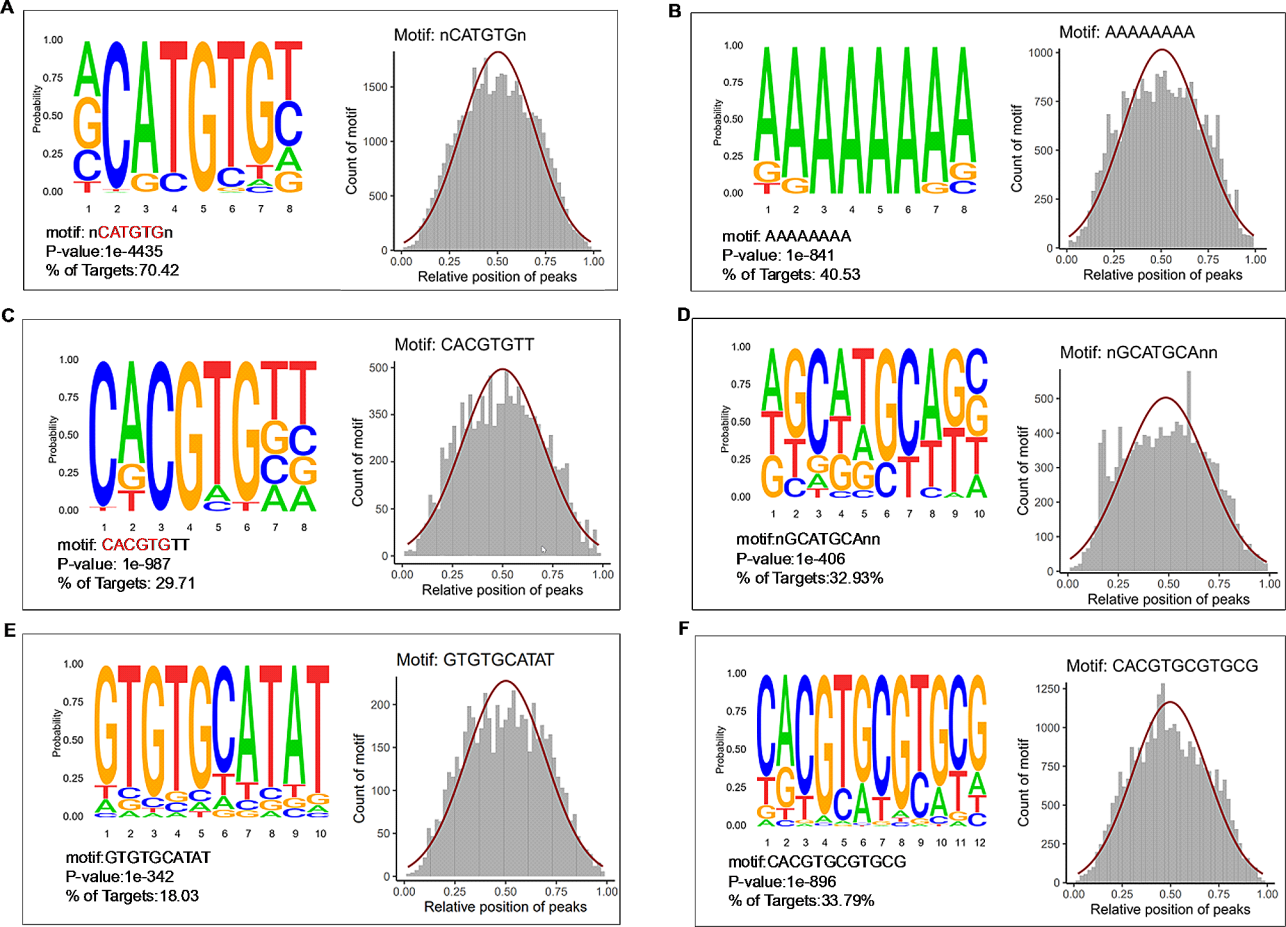
The main motifs bound by ZmMYC2. The main motifs were detected by DAP-seq to be bound by ZmMYC2 with length of 8 (**A**-**C**), 10 (**D**-**E**) and 12 (**F**). % of Target is percentage of target sequences with motif. The core sequences of E-box and G-box were labeled in red (A, C). Normal distribution of motifs is shown to reveal motif centrality. The x-axis indicates the relative position of motif in the peak. The y-axis indicates the count of motif on the different position of peak. Gaussian fitting curve is highlight in dark red

**Fig. 7:**
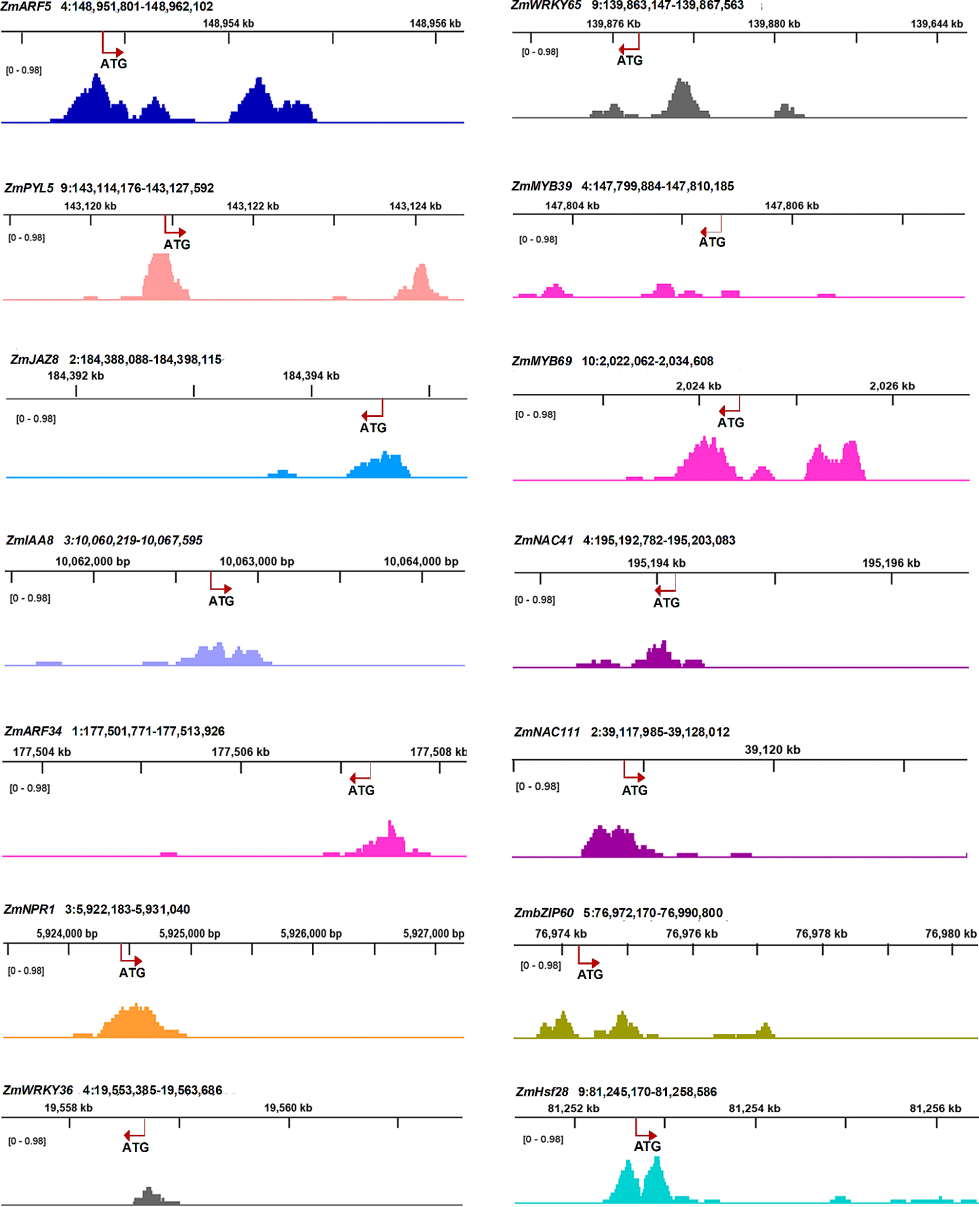
Motif distribution bound by ZmMYC2 on selected genes Gene location on maize chromosomes are shown with gene names. The initial codon and transcription direction are indicted with the red arrow. The peaks indicate the binding of ZmMYC2 on genes by DAP-seq analysis with fold enrichment

ZmMYC2 belongs to the bHLH family that can recognizes and binds to *cis*-elements like the G-box, E-box and CAnnTG [65, 66]. DAP-seq analysis revealed that ZmMYC2 bound to abundant and diverse target motifs. The motifs with percentage of target sequences more than 10% were shown in Fig. 6, including the 8 bp motifs nCATGTGn (70.42%), AAAAAAAA (40.53%), CACGTGTT (29.71%); the 10 bp motifs nGCATGCAnn (32.93%), GTGTGCATAT (18.03%); the 12 bp motif CACGTGCGTGCG (33.79%). Among these motifs, the classic E-box and G-box sequence were also detected (Fig. 6A and C). Centrality analysis indicated that these motifs were located in the corresponding peaks detected by DAP-seq in the pattern of normal distribution and with peak value of ∼ 0.5, suggesting as the potential *cis*-elements of ZmMYC2. In addition, we also analyzed the binding sites of ZmMYC2 on some TRGs and found that most binding sites for these genes are located on promoters (Fig. 7). However, binding on the gene exon or intron or 3’ UTR was also observed, for example, *ZmARF5*, *ZmPYL*, *ZmMYB69*. All these results indicate that ZmMYC2 might conduct functions through acting on diverse *cis*-elements.

## Discussion

MYC2 as the master regulator of JA signaling plays important and diverse roles in plant growth and environmental responses. Here we explored the regulatory target genes of ZmMYC2 using the convenient protoplast transient expression system and revealed abundant TRGs and IRGs of ZmMYC2 with diverse target *cis*-elements in maize, providing the basis for further functional characterization of ZmMYC2 and target genes.

MYC2 has been reported to bind to the promoters of *PLT1* and *PLT2* and repress their gene expression, resulting in negative regulation on root stem cell and meristem activity [67]. ERF115 was involved in cell division in quiescent center and positively regulated by JA through MYC2, which promoted stem cell and regeneration after root damage [68]. Auxin synthesis genes *Yucca6* and *Yucca9* participated into root geotropism and lodging resistance in maize [69]. All these genes were found in our study to be the target genes of ZmMYC2, indicating involvement of ZmMYC2 in root development through regulating these genes. In addition, MYC2 also works in leaf senescence. MYC2 inhibited *CAT2* gene expression, leading to H_2_O_2_ accumulation and upregulation of senescence associated genes *SAG12*, *SAG13*, *SAG29*, *SAG113* and repression of photosynthesis related genes *CAB1* and *RBCS* [70, 71]. JA induced senescence related gene *Dof2.1* through MYC2 and Dof2.1 also activated MYC2 promoter, forming the positive feedback loop in leaf senescence [72]. During rape leaf senescence, MYC2 was activated by ABA and further elevated starch degrading genes *AMY3*, *BAM1* and sugar transporting gene *SUT1*, S*UT4* and *SWEET11* [73]. In tomato, SlMYC2 increased expression of chlorophyll degradation gene *SIPAO* and promoted senescence [74]. In our results, we also found many senescence associated genes to be the target regulating genes of ZmMYC2 (Table S13), suggesting the function of ZmMYC2 in maize senescence regulation and providing basis for such investigation in the future.

MYC2 mediates plant hormone signaling crosstalk among JA, ABA, ethylene, GA and SA to widely involve in various biological processes [75]. In Arabidopsis, AtMYC2 bound to promoters of a number of defense related genes to positively regulate *MKK4*, *RIN4*, *ICS1* and negatively regulate PEPR1, then playing dual roles in PTI and ETI through mediating SA bisoynthesis and signaling [76]. AtMYC2 also increased GA inactivation genes to inhibit endogenous GA level and plant growth through JA signaling [77]. MYC2 was also involved in drought response through activating *RD22* and *ADH*, genes in ABA signaling pathway [78, 79]. Auxin synthesis was negatively regulated by MYC2 through inhibiting tryptophan biosynthesis, leading to repression of leaf vein development [80]. In this study, many hormone related genes were identified as the target regulating genes of ZmMYC2, indicating the important role of ZmMYC2 in hormone signaling crosstalk in maize, which could be characterized in the future investigation.

MYC2 mediates JA signaling to participate into biotic and abiotic stress responses. Overexpression of MYC2 in rice increased *PR* gene expression to confer resistance to bacterial blight [81]. In Arabidopsis, MYC2 interacted with EIN3 to regulate *ERF* gene expression and enhance *PDF1.2* expression in response to pathogen infection [18]. Here we observed a number of *PR* genes to be upregulated by transient overexpression of ZmMYC2 in maize protoplasts, suggesting potential involvement of ZmMYC2 in disease resistance. In addition, MYC2 in apple bound to the G-box of *MdCBF1* gene promoter to regulate cold tolerance [82]. In wheat, MYC2 regulated melatonin synthesis gene *ASMT* to improve drought resistance [83]. In Arabidopsis, MYC2 inhibited *CAT2* gene expression and promoted H_2_O_2_ accumulation by JA and leaf senescence, resulting in lower salt tolerance [28, 70]. ZmMYC2 was also identified to regulate many abiotic stress response genes in this study (Table S13), implicating participation of ZmMYC2 in these processes.

MYC2 has been demonstrated to involve specialized metabolism regulation. At MYC2 directly bound to gene promoters of terpene synthases to increase terpene accumulation for defense to *Bemisia tabaci* [84]. In *Tripterygium wilfordii*, TwMYC2a and TwMYC2b negatively regulated gene expression of TwTPS27a/b and triptolide biosynthesis [85]. In maize, CUT&Tag-seq and RNA-seq analysis revealed that ZmMYC2 directly targeted and regulated *ZmIGPS1/3* and *BX10/11/12/14* in benzoxazinoid metabolism [31]. In this study, many benzoxazinoid related genes including *ZmIGPS1*, *ZmBX4*, *ZmBX5*, *ZmBX6*, *ZmBX12* and *ZmBX13* (Table S13), among which *ZmIGPS1* and *ZmBX12* have been demonstrated to be the target genes of ZmMYC2 [31], consistent with the previous study. In addition, ZmMYC2 was also reported to regulate terpenoid phytoalexin biosynthesis [30]. Here we detected *ZmKSL2* (Zm00001d041082) as the target genes of ZmMYC2, which further validates our data accurancy. Furthermore, MYC3, the homolog of MYC2, dominantly affected flavonoid biosynthesis and cotton bollworm resistance [86]. Many flavonoid synthesis genes were also detected as the target regulating genes of ZmMYC2 (Table S13), suggesting the potential role of ZmMYC2 in maize flavonoid biosynthesis regulation.

MYC2 is usually reported to bind *cis*-elements like the G-box (CACGTG) and the E-box (ACGT or CANNTG) to regulate downstream target gene expression [65, 66]. For instance, MYC2 bound the G-box on target gene promoter to activate JA related gene expression and positively regulated JA signaling transduction and corresponding defense response [87, 88]. In *Artemisia annua*, AaMYC2 bound to the G-box like of *CYP71AV1* and *DBR2* promoters, two key genes of artemisinin biosynthesis, to elevate their expression [89]. SmMYC2 bound to the E-box on *SmCYP98A14* gene promoter to regulate gene expression and tanshinone biosynthesis [66, 90]. In addition to G-box and E-box, we also found many new *cis*-elements to be recognized by ZmMYC2, which should be characterized in the future investigation about their functions and mechanisms.

We also found that ZmMYC2 regulated a lot of genes with various function annotations. Fox example, many glycosyl transferase encoding genes were observed as the TRGs of ZmMYC2 (Table S14). Rice glycosyl transferase UGT2 is regulated by bZIP23 to elevate salt tolerance [91]. UDP-glucose transferase EDR1 is the pivotal factor for endosperm development of rice [92]. In maize, the UDP-glucose transferase encoding gene *sk1* is involved in pistil protection through blocking JA accumulation [93]. In addition, many glycoside hydrolase encoding genes were also regulated directly by ZmMYC2, which might be involved in specialized metabolism. In tartary buckwheat, FtGH1 (β-glucosidase) converted rutin to quercetin, playing the key role in rutin hydrolysis [94]. Glutathione S-transferase is a super family with the main functions of detoxification and specialized metabolism. The glutathione S-transferase encoding genes were also regulated by ZmMYC2. In tea tree, CsJAZ1-CsMYC2.2 mediated JA signaling to regulate CsGSTU45 for H_2_O_2_ accumulation, affecting resistance to *Colletotrichum camelliae* infection [95]. SWEET protein is the sugar transporter. In barley, SWEET11b is responsible for transportation of sugar and cytokinin [96]. SWEET17 in Arabidopsis regulated lateral root growth and drought resistance [97]. Many SWEET genes were also found to be TRGs of ZmMYC2, indicating participation of ZmMYC2 in regulating these processes.

Moreover, many genes encoding cytochrome P450, pentatricopeptide repeat (PRR) and protein kinase were identified to be regulated by ZmMYC2 with promoter binding (Table S14). These genes are widely involved in plant growth, development and stress response, suggesting the various functions of ZmMYC2. OsNBL3, the mitochondrion localized PRR, played a role in intron splicing of *nad5* to increase stress response in rice [98]. Maize PRR genes regulated kernel size and yield determination [99, 100]. Protein kinases like LRR receptor-Like protein kinase, serine/threonine-protein kinase and calcium-dependent protein kinase function in most biological processes of plant life [101–103]. Although we observed regulation of these genes by ZmMYC2 here, the regulatory function and mechanism should be validated in the future investigation to be linked with specific biological process.

## Conclusions

**Fig. 8:**
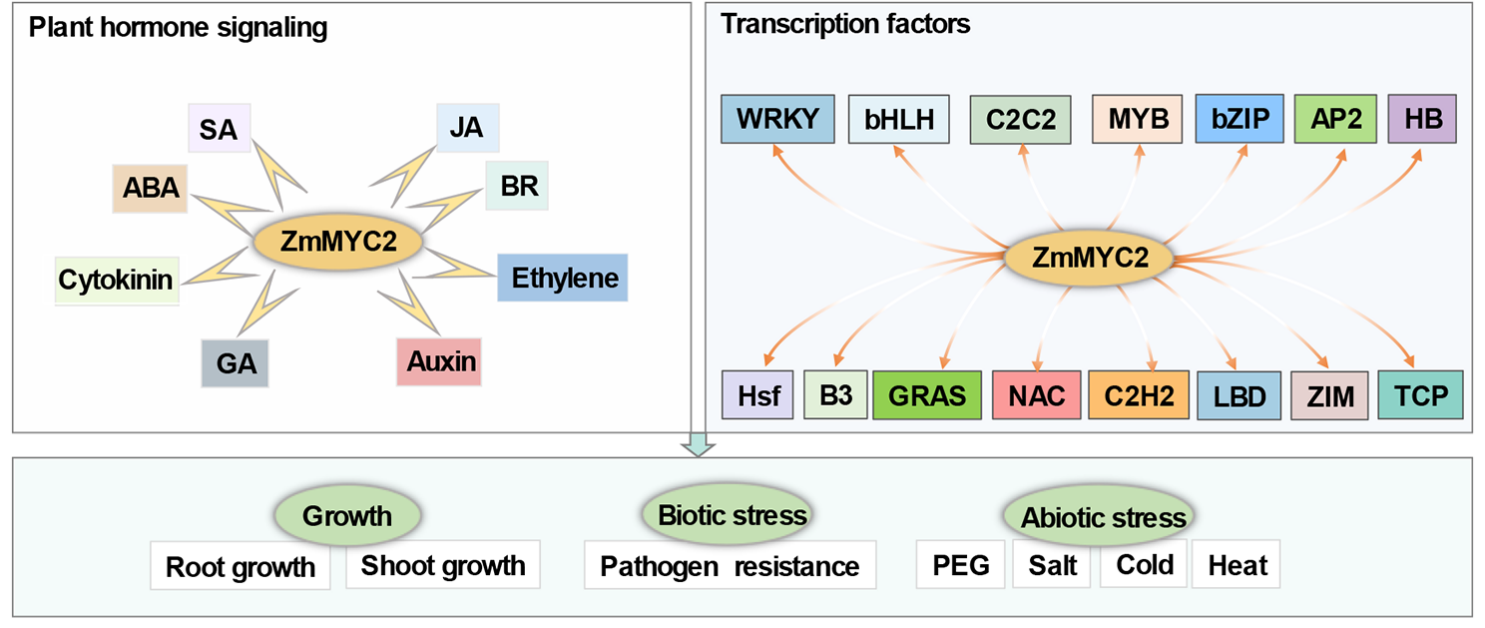
The proposed regulating model of ZmMYC2 ZmMYC2 targets to many plant hormone signaling genes and transcription factor encoding genes, which confers the broad function of ZmMYC2 in plant growth and stress responses through regulating these genes in various pathways

MYC2 not only plays as the master regulator of JA signaling, but also functions in many other biological processes in plants. Here we combined RNA-seq and DAP-seq data to explore the target regulating genes of ZmMYC2. A lot of genes were identified to regulated by ZmMYC2 through direct binding with various function annotation. Meanwhile, many new *cis*-elements were identified to be recognized and bound by ZmMYC2 besides the classic G-box and E-box. This investigation indicates the broad functions of ZmMYC2 through targeting diverse pathway genes including multiple plant hormone signaling genes and many transcription factor to participate various processes in plant growth and environmental responses (Fig. 8). Our study provides the important data basis for further function and mechanism characterization to clarify the complex signaling transduction and regulation in maize.

## Materials and methods

### Plant materials

Seeds of maize inbred line Mo17 were obtained from Maize Research Institute in Sichuan Agricultural University and germinated and grown in the growth chamber with the condition of 16 h light and 8 h dark at 28 °C. The maize seedling with two leaves were transferred into the darkness for 5 d until etiolation, ready for protoplast preparation.

### Protoplast preparation and transfection

Maize leaf protoplast isolation was conducted as described previously [104]. ZmMYC2 was ligated into pBI221 and transfected into maize protoplasts as described previously [30], the empty pBI221 vector was used as the control. Specifically, 20 μg plamids were mixed with 200 μL protoplasts and 220 μL transfection buffer (40% PEG4000, 0.2 M mannitol, 0.1 M CaCl_2_) and kept in the darkness for 15 min. 1 mL W5 solution (154 mM NaCl, 125 mM CaCl_2_, 5 mM KCl and 2 mM MES) was added to terminate transfection. Protoplasts were collected with gentle centrifuge and resuspended into 500 μL W5 solution and incubated in the dark at 25 °C for 16–24 h, ready for protoplast collection and RNA extraction.

### RNA extraction and RNA-seq analysis

RNA extraction from maize protoplasts was performed as described previously [64]. The HiPure HP Plant RNA Mini Kit (Magen Biotech, Guangzhou, China) was used to extract RNA from maize protoplasts following the manufacture protocol. RNA-seq analysis was conducted by Novogene (https://www.novogene.com/). cDNA library sequencing and data filtering were conducted on an Illumina NovaSeq 6000. Specifically, transcriptome libraries were prepared using a TruSeqTM RNA sample preparation kit from Illumina (San Diego, CA, USA). Paired-end libraries were sequenced by Illumina NovaSeq6000 sequencing. Deferentially expressed genes (DEGs) were identified using the DESeq2 software [105]. GO and KEGG enrichment were analyzed by GSEA (https://www.gsea-msigdb.org/gsea/index.jsp) [105, 106].

### Quantitative PCR analysis

cDNA was prepared using the total RNA extracted above with the reverse transcriptase kit (Vazyme Biotech, Nanjing, China). Quantitative PCR (qPCR) analysis was conducted using the SYBR GREEN qPCR Master MIX (Vazyme Biotech) on the Bio-Rad CFX96 as described previously [64]. The maize gene *ZmEf1a* was used as the endogenous control according to previous study [64].

### DAP-seq analysis

The maize gDNA was extracted from the two-week-old maize Mo17 leaves with the CTAB reagent. The gDNA was broken into fragments with 200–500 bp by ultrasonication for further binding. The recombinant ZmMYC2 protein was prepared and used to incubate with the fragmented gDNA as described previously [29]. The binding gDNA of ZmMYC2 was extracted with the mixture of chloroform and isopentanol (24:1, v/v) and re-purified for sequencing. DAP-seq analysis was conducted and reported in our previous study [29]. Peak analysis of promoter was carried out by IGV-sRNA [107, 108].

### Centrality analysis of motifs

The main motifs detected by DAP-seq were located in the peaks by the Homer software to acquire the motif position and peak length (http://homer.ucsd.edu/homer/introduction/update.html). The relative position of motif in peaks was shown in the histogram by the ggplot2 software (http://www.sthda.com/english/wiki/ggplot2-barplots-quick-start-guide-r-software-and-data-visualization). All motif and peak information were included the raw data of DAP-seq.

### Gene expression analysis

Maize gene expression data under different growth periods and stress treatment including PEG, Salt, Heat, Cold, infection of *Colletotrichum graminicola* and *Cercospora zeina* were obtained on the website of Maize eFP Browser (https://bar.utoronto.ca/) [109]. RNA-seq data analysis was conducted on ChiPlot (https://www.chiplot.online/#) and Bioinformatics (https://bioinformatics.com.cn/) [107, 108].

### Electronic supplementary material

Below is the link to the electronic supplementary material.

Supplementary Material 1

Supplementary Material 2

Supplementary Material 3

Supplementary Material 4

Supplementary Material 5

Supplementary Material 6

Supplementary Material 7

Supplementary Material 8

Supplementary Material 9

Supplementary Material 10

Supplementary Material 11

Supplementary Material 12

Supplementary Material 13

Supplementary Material 14

Supplementary Material 15

## Data Availability

DAP-seq and RNA-seq raw data were deposited into Genome Sequence Archive of CNCB-NGDC (https://ngdc.cncb.ac.cn/) with the accession ID of PRJCA022022 and PRJCA022023, respectively.
