# Correction: Genome-wide identification of ZmMYC2 binding sites and target genes in maize

**DOI:** 10.1186/s12864-024-10386-z

**Published:** 2024-05-17

**Authors:** Lijun Liu, Yuhan Zhang, Chen Tang, Jine Wu, Jingye Fu, Qiang Wang

**Affiliations:** 1 State Key Laboratory of Crop Gene Exploration and Utilization in Southwest China, College of Agronomy, Sichuan Agricultural University, Chengdu, 611130 China; 2 College of Life Science, Sichuan Agricultural University, Yaan, 625014 China

**Correction: BMC Genomics 25, 397 (2024)**

**https://doi.org/10.1186/s12864-024-10297-z**

Following publication of the original article [1], we have been notified that the y-axis labels in Figs. 1, 2, 3 and 8 had been mirrored and made unreadable.

The original article has been corrected.
