# Supplementary material for: Genome-wide identification of ZmMYC2 binding sites and target genes in maize
Source: BMC Genomics. 2024 Apr 23;25:397. doi: 10.1186/s12864-024-10297-z (PMC11036654; doi:10.1186/s12864-024-10297-z)
Supplement: Supplementary file 15 — Supplementary Material 15 [file 12864_2024_10297_MOESM15_ESM.pdf]

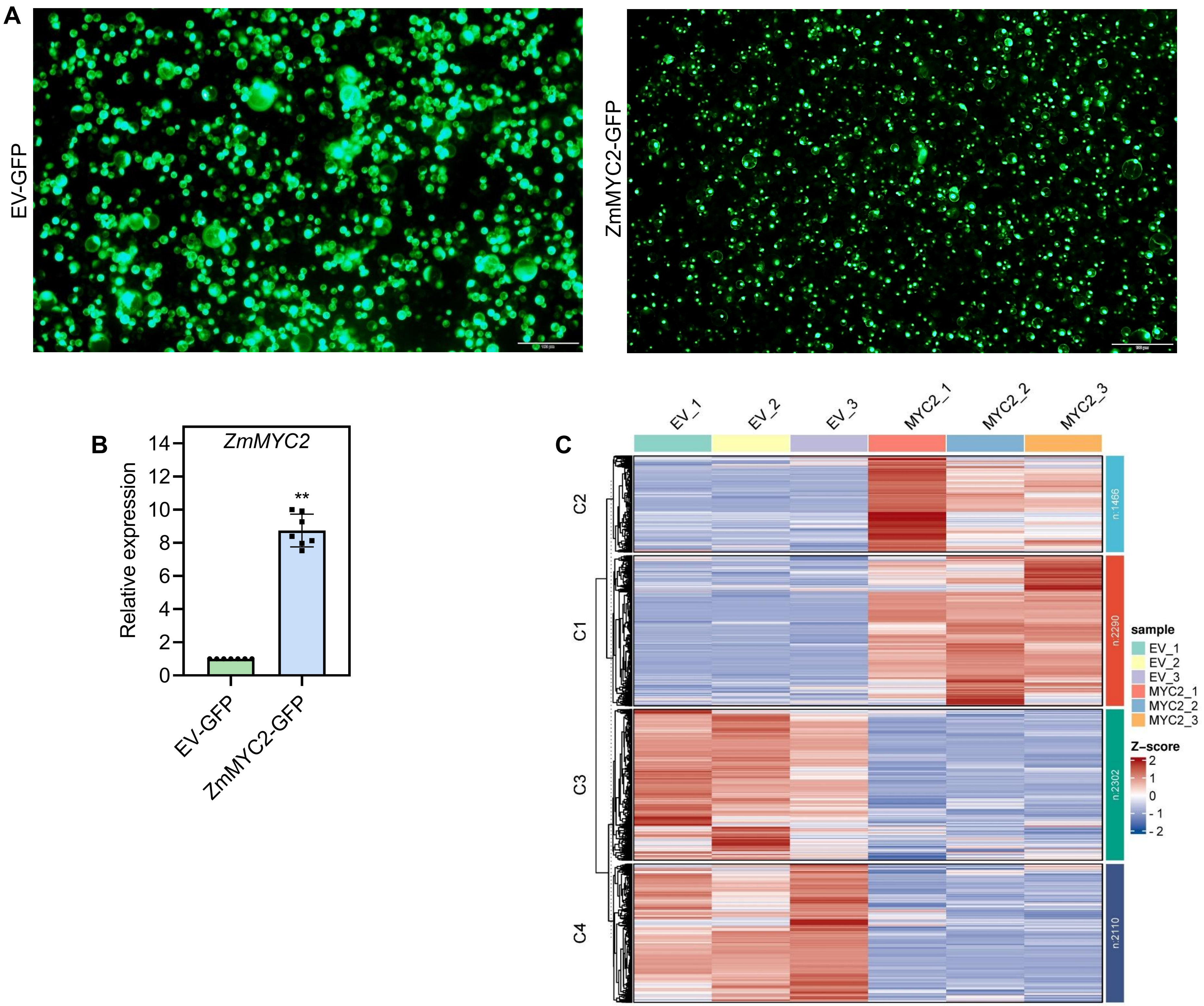

Figure S1. Transient overexpression of ZmMYC2 in maize protoplast for RNA-seq analysis. (A) Maize protoplasts with transient overexpression of the empty verctor and ZmMYC2. Both of them carried the GFP tag. (B) Gene expression of ZmMYC2 after overexpression in maize protoplasts. EV, the empty vector. * *$P < 0.01$ (Student's *t*-test). (C) RNA-seq heatmap trend GO pathway analysis to show repeatability among for different replicates.
